# Assessing the impacts of inter-basin water transfer projects on ecosystem services in water source areas: Evidence from the Hanjiang River Basin

**DOI:** 10.1371/journal.pone.0323068

**Published:** 2025-05-30

**Authors:** Nana Zhuang, Min Wang, Chenyi Shi, Shen Fu, Qiyuan Yang, Conghui Ding, Yiao Ouyang, Hai Liu

**Affiliations:** 1 Faculty of Resources and Environmental Science, Hubei University, Wuhan, China; 2 Research Center of Territorial Space Management, Hubei University, Wuhan, China; 3 Hubei Key Laboratory of Regional Development and Environmental Response, Hubei University, Wuhan, China; Gujarat Institute of Desert Ecology, INDIA

## Abstract

Inter-basin water transfer projects (IBWT) are a key strategy for alleviating regional water shortages. However, studies on the long-term effects of such projects on ecosystem services (ESs) in water source areas, as well as their spatiotemporal evolution, remain insufficient. In particular, the specific impacts of the entire project lifecycle (project initiation, dam heightening, project operation, and ecological restoration) on ESs need further exploration. This study focuses on the Middle Route of the South-to-North Water Diversion Project in China, utilizing multi-source datasets (land use and land cover, meteorological data, soil texture, digital elevation models, normalized difference vegetation index, and net primary productivity), applying the biophysical model method to examine the spatiotemporal variations in ESs across the Hanjiang River Basin over the past three decades, and investigates the impact of IBWT on the ESs of water source areas. The findings reveal: (1) During the project initiation phase (2000–2010), vegetation restoration strategies enhanced soil retention by over 60%, demonstrating that simultaneous project-ecological implementation can mitigate habitat degradation risks. (2) The dam heightening phase (2005–2010) drove spatial reconfiguration of water-energy fluxes, leading to declines in water conservation and carbon sequestration in the midstream region, while enhancing flood mitigation and climate regulation in the reservoir area, unveiling the reshaping mechanisms of dam heightening on ESs supply patterns. (3) The operation phase exhibited significant temporal heterogeneity: initial operation (2010–2015) saw a sharp decline in water conservation (>40%) and soil retention (>60%) due to hydrological disturbances, whereas sustained operation (2015–2020) restored water conservation by nearly 70% and soil retention by over 40% through ecological restoration, alongside a net increase of 14.14% in carbon sequestration, confirming the time-lag compensation effects of restoration measures and the dynamic interplay between ecological restoration and project interventions. This research presents empirical evidence supporting the sustainable management and ecological restoration of IBWT, emphasizing the need to balance spatial water allocation with ecological conservation.

## 1. Introduction

Global water resources are unevenly distributed, with some regions facing severe water scarcity issues. With the rapid acceleration of population growth, industrialization, and urbanization, the demand for water is steadily increasing, particularly in economically developed and densely populated areas, where water scarcity has become increasingly prominent [1]. To overcome this challenge, inter-basin water transfer projects (IBWT) have been implemented worldwide to redirect water from regions with abundant supplies to those facing scarcity, thereby meeting the growing regional water demands [2]. Notable examples include the United States’ Central Valley Project (CVP) and California State Water Project (SWP), Canada’s Churchill River Diversion (CRD), Australia’s Snowy Mountains Scheme, and China’s South-to-North Water Diversion Project (SNWD). Additionally, IBWT in Europe, such as the Tagus-Segura Water Transfer in Spain and the Provence-Alpes-Côte d’Azur Water Transfer in France, have achieved significant success. Through effective water resource redistribution, these projects have ensured agricultural production, industrial development, and urban water supply, thus fostering long-term economic stability and societal progress in these countries [3].

Ecosystem services (ESs) have become a critical metric for assessing the impact of human activities on the natural environment, particularly within the framework of global environmental governance and sustainable development. ESs refer to the various functions provided by the natural environment, which support or enhance human well-being, either directly or indirectly [4]. IBWT as large-scale water infrastructure projects, involve complex water resource management. These projects often alter hydrological characteristics such as water flow speed, discharge, and water levels, which can disrupt the natural hydrological processes and ecological balance of water source areas [5]. Existing studies have shown that these hydrological changes can have profound effects on ecosystem stability and function, potentially leading to ecological degradation and the loss of biodiversity [6]. For instance, the CRD resulted in increased water temperatures and decreased water transparency in Southern Indian Lake, exacerbating eutrophication and subsequently impacting aquatic plant development and fish reproduction [7]. Similarly, the CVP and SWP modified the hydrological dynamics of the Sacramento-San Joaquin River Delta, causing significant wetland habitat loss, the disappearance of certain native species, and a notable reduction in plankton and benthic invertebrate diversity and abundance [8]. Consequently, thorough investigation into the long-term effects of IBWT on the ESs of water source regions is crucial. Additionally, developing robust management strategies is necessary to promote sustainable water resource use while safeguarding the ecological functions of these areas.

Large areas of forest and cropland typically submerge during the construction and operation of IBWT, leading to the decomposition of vast amounts of organic matter and the release of greenhouse gases, which significantly reduces carbon storage capacity [9] Furthermore, such projects affect the hydrological cycles and water quality of rivers, exerting pressure on both the reservoir area and downstream ecosystems, particularly in terms of habitat quality and biodiversity [10]. Briones-Hidrovo et al. [11] pointed out that although IBWT enhances the water regulation and flood control of reservoirs to some extent, the resulting water quality degradation, biodiversity loss, and habitat function decline ultimately lead to the overall degradation of ESs in the reservoir area. Notably, current research mostly focuses on impact analyses during specific stages or in localized areas, emphasizing changes over time, and lacks a systematic assessment of the spatiotemporal evolution of ESs throughout the entire lifecycle of IBWT (including project initiation, dam heightening, project operation, and ecological restoration). Therefore, in-depth studies on the spatiotemporal changes of ESs across the lifecycle of such projects are urgently needed to provide theoretical foundations and enhance decision-making processes for their scientifically informed planning and management.

The methods for assessing ESs primarily include the equivalent factor method and the biophysical model method. The equivalent factor method is a relative assessment approach based on per-unit-area ecosystem service factors, with its core idea being the quantification of services from different ecosystems through standardized factors. This method is straightforward to implement and requires minimal data, making it suitable for large-scale or preliminary ESs assessment [12]. In contrast, the biophysical model method is a more refined and complex method that simulates and calculates the physical, chemical, and biological processes of ecosystems, enabling a more precise description of the dynamic changes and spatiotemporal heterogeneity of ESs [13]. The biophysical model method typically relies on multi-source datasets, including land use and land cover (LULC), precipitation, temperature, soil texture, digital elevation models (DEM), normalized difference vegetation index (NDVI), and net primary productivity (NPP). These datasets provide critical input parameters for the models, supporting comprehensive ESs assessment. For instance, Liu and Feng et al. [14,15] applied the biophysical model method, integrating LULC, NDVI, and NPP data, to evaluate ESs in the Yellow River Basin and the Loess Plateau, revealing spatiotemporal variations and examining the driving effects of climate change, ecosystem structure, and landscape configuration on ESs. Therefore, this study will adopt the biophysical model method to evaluate the ESs of the Hanjiang River Basin (HJRB) in order to explore their spatial heterogeneity and the underlying mechanisms.

The SNWD is the largest IBWT in the world. Among its components, the Middle Route of SNWD (MR-SNWD) is responsible for diverting water resources from the HJRB to North China. Since its official operation in 2014, the MR-SNWD has transferred over 65 billion m³ of water, benefiting 108 million people and significantly contributing to the economic development of North China. However, the implementation of the MR-SNWD has introduced significant challenges to the ecological integrity of the water source regions. Specifically, the project has modified both the flow volume and velocity in the Hanjiang River’s middle and downstream sections, resulting in decreased water quality stability and adversely affecting aquatic biodiversity and fish community composition [16]. Furthermore, Zhang et al. [17] pointed out that large-scale water diversion has contributed to reductions in both water yield and carbon sequestration within the HJRB. Therefore, conducting in-depth research on the long-term impacts of the MR-SNWD on ESs in the HJRB and exploring the patterns of ESs evolution throughout its entire lifecycle is of significant scientific value and practical importance. This research not only provides a scientific basis for ecological conservation in the HJRB but also addresses gaps in existing studies by revealing the dynamic mechanisms through which IBWT influences ESs across its entire lifecycle. Specifically, this study quantifies the reshaping effects of different project phases on ESs supply patterns and proposes management strategies based on the dynamic interplay between ecological restoration and project interventions. These findings offer a scientific foundation for optimizing the balance between water resource redistribution and ecological protection while also providing valuable insights and methodologies for assessing ecological impacts and ensuring sustainable management of other IBWT projects worldwide.

This study utilizes the MR-SNWD as a case to systematically evaluate its effects on ESs in the HJRB through the biophysical model method. The aim is to reveal the enduring impacts of IBWT on ESs in water source areas and to understand their spatiotemporal evolution. Specifically, the main aims of this research are outlined as follows: (1) analyzing the temporal and spatial variations in ecosystem structure and ESs of the HJRB from 1990 to 2020; (2) conducting a comparative analysis of the differences in the ecosystem structure of water source areas before and after the initiation of the project; (3) investigating the spatiotemporal effects of IBWT on the ESs of water source areas; (4) drawing on the principles of ESs across various ecosystems, and in conjunction with an analysis of the implementation of the MR-SNWD, proposing scientifically sound and practical strategies for ecological restoration and sustainable management.

## 2. Materials and methods

### 2.1 Study area

The MR-SNWD represents a significant infrastructure launched by the Chinese government in 2003, aimed at effectively alleviating the water scarcity issues faced by North China. Located in Nanyang, Henan Province, and Shiyan, Hubei Province, the Danjiangkou Reservoir functions as the primary water source for the project [18]. The Danjiangkou Dam heightening project, undertaken between 2005 and 2010, aimed to increase the reservoir’s storage capacity. This modification involved raising the dam’s elevation from 162 m to 176.7 m, which subsequently expanded the reservoir’s volume from 17.4 billion m³ to 29.05 billion m³. Since December 12, 2014, the MR-SNWD has been officially operational, providing a stable water supply for North China. Additionally, to mitigate the environmental impacts of the MR-SNWD on the midstream and downstream regions of the HJRB, the Yangtze–Hanjiang Water Diversion Project (YHWD) has been channeling water to the downstream region of Xinglong Reservoir since September 26, 2014, providing an average yearly volume of 3.7 billion m³ [19].

**Fig 1:**
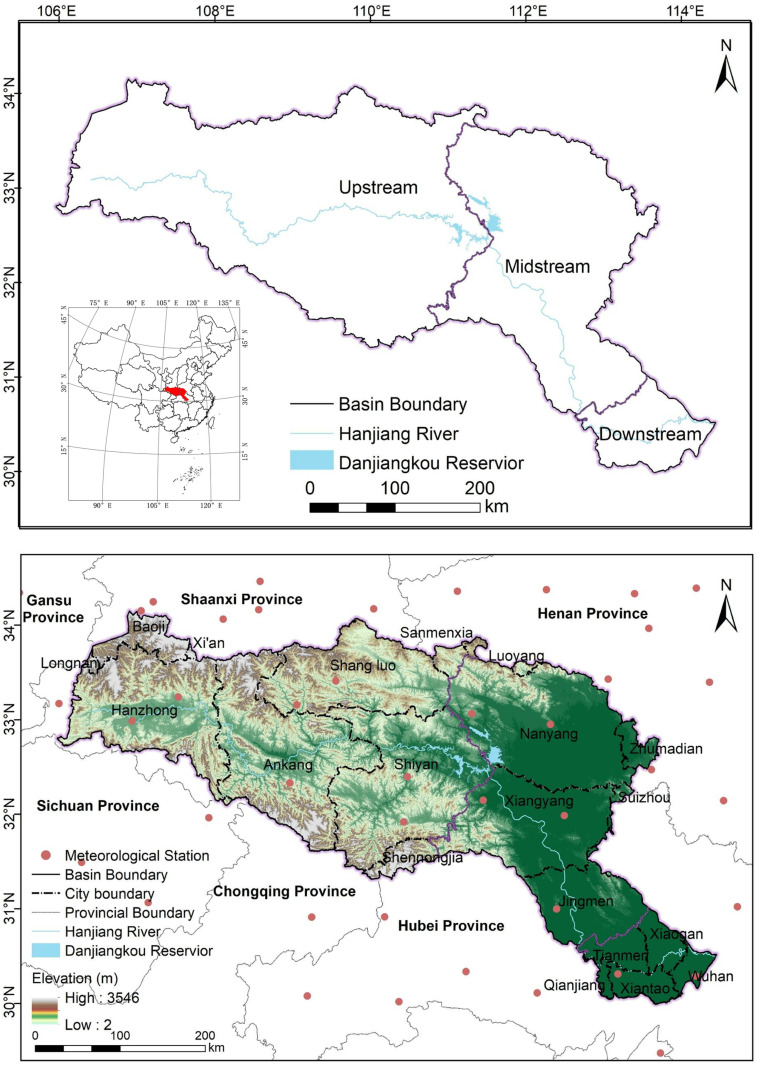
The HJRB’s geographic position and elevation.

The HJRB, serving as the water source for the MR-SNWD, extends across several provinces, including Shaanxi, Henan, and Hubei, covering an area of 159,000 km². Geographically, it lies between longitudes 106°E and 114°E, and latitudes 30°N and 34°N (Fig 1) [20]. The basin’s terrain gradually slopes from west to east, and it is typically divided into upstream, midstream, and downstream areas. It spans the second and third major geological structures of China, resulting in diverse topographical features, including undulating mountains, hills, and flat plains. Characterized by a subtropical monsoon climate, the region exhibits an annual temperature range between 15°C and 17°C, coupled with an average yearly precipitation of 873 mm.

### 2.2 Data sources

This research employs several datasets, encompassing LULC, meteorological data, soil texture, DEM, NDVI, and NPP. All datasets were converted into the CGCS2000GKZone_19 geographic coordinate system and resampled to a constant spatial resolution of 250 meters in order to preserve homogeneity. Table 1 contains a list of these datasets’ comprehensive sources.

**Table 1:** Summary of the primary data.

Data	Date source/ processing	Accessibility
LULC	Wuhan University [21]	Available upon request from Wuhan University.
meteorological data (precipitation and temperature)	National Meteorological Science Data Center, http://data.cma.cn/	Publicly accessible.
Soil texture (sand, silt, clay, and organic matter content in soil)	National Tibetan Plateau Scientific Data Center, https://data.tpdc.ac.cn/	
DEM	National Aeronautics and Space Administration (NASA), https://search.earthdata.nasa.gov/	
NDVI	NDVI data for the period 1990–2020 were retrieved using the Google Earth Engine (GEE) platform, leveraging imagery from the Landsat 5/7/8 satellites.	Publicly accessible via Google Earth Engine.
NPP	On the GEE platform, utilizing the CASA model in conjunction with TerraClimate, Landsat 5/7/8, and LULC, the NPP from 1990 to 2020 was obtained.	

#### 2.3 ESs assessment.

**Fig 2:**
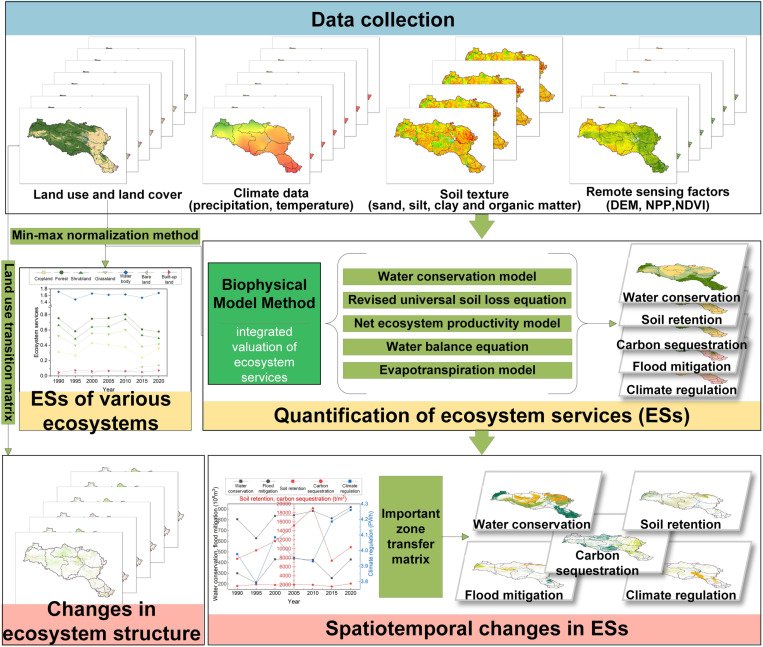
Workflow diagram for ESs assessment.

IBWT alters hydrological processes and can have profound impacts on the ESs of water source areas. These projects typically affect groundwater recharge and surface water regulation by modifying flow rates, velocities, and directions [22]. Such changes may exacerbate soil erosion, lead to water and soil loss, and undermine vegetation stability [23]. Additionally, the dam heightening project raises the reservoir’s water level and capacity, increasing the water surface area, which in turn alters evaporation rates and influences local climate. At the same time, the reservoir’s water storage and scheduling capacities are enhanced, further improving flood control and water allocation [24]. Based on these potential impacts, this study focuses on assessing five key ESs: water conservation (WC), soil retention (SR), flood mitigation (FM), carbon sequestration (CS), and climate regulation (CR). To clearly illustrate the analytical methods and steps of this study, Fig 2 presents the complete workflow from data collection to ESs assessment.

#### 2.3.1 Water conservation.

WC refers to the ecological function whereby ecosystems intercept and detain precipitation, enhancing soil infiltration and storage, conserving soil moisture, regulating runoff, and replenishing groundwater, thereby increasing the availability of water resources. This study employs the water conservation model utilized in the China Ecosystem Assessment Framework by Ouyang et al. [25], which calculates WC based on precipitation, temperature, and LULC.


$${\text{Q}}_{\text{wr}}\text{=S×(P-R-E)×}{\text{10}}^{\text{-3}}$$ (1)

where ${\text{Q}}_{\text{wr}}$ is the amount of WC (m³/a); S is the pixel area (m²); P refers to the annual precipitation (mm/a); R indicates the surface runoff on an annual basis (mm/a); E signifies the annual evaporation (mm/a).

#### 2.3.2 Soil retention.

SR encompasses the ability of ecosystems, including forests and grasslands, to mitigate the erosive impact of rainfall. This function is facilitated through multiple layers, such as the canopy, litter, and root systems, thereby enhancing soil resistance to erosion and reducing soil loss. This study uses data on precipitation, soil properties, DEM, NDVI, and LULC to quantify SR using the Revised Universal Soil Loss Equation (RUSLE) model [26].


$${\text{Q}}_{\text{sr}}\text{=R×K×L×S×(1-C×}{\text{PR}}_{\text{i}}\text{)}$$ (2)

where ${\text{Q}}_{\text{sr}}$ represents the amount of SR (t/hm²·a); R represents the rainfall erosivity (MJ·mm/hm²·h·a); K indicates soil erodibility (t·hm²·h/hm²·MJ·mm); the slope length factor is denoted by L; S corresponds to the slope gradient factor; C refers to the crop management factor; ${\text{PR}}_{\text{i}}$ signifies the support practice factor.

#### 2.3.3 Carbon sequestration.

CS describes the role of ecosystems in absorbing atmospheric carbon dioxide, converting it into organic matter and storing carbon within plants and soils. This study utilizes the net ecosystem productivity model [27] to calculate CS based on NPP, temperature, and precipitation.


$$\begin{array}{c} {\text{Q}}_{\text{t}{\text{CO}}_{\text{2}}}\text{=}\frac{{\text{M}}_{{\text{CO}}_{\text{2}}}}{{\text{M}}_{\text{C}}}\text{×(NPP-}\sum_{\text{i=1}}^{\text{12}}\text{0.22}{\text{(e}}^{\text{0.0912}{\text{T}}_{\text{i}}}\text{+ln}\left(\text{0.3145}{\text{P}}_{\text{i}}\text{+1}\right)\text{)×30×46.5\textbackslash{}\%}) \end{array}$$ (3)

where ${\text{Q}}_{\text{t}{\text{CO}}_{\text{2}}}$ is the amount of CS (gCO2/m²a); $\frac{{\text{M}}_{{\text{CO}}_{\text{2}}}}{{\text{M}}_{\text{C}}}$ is the coefficient for converting CO_2_ to C, which is $\frac{\text{44}}{\text{12}}$; NPP is net primary productivity (gC/m²a); ${\text{T}}_{\text{i}}$ refers to the monthly average temperature (°C); ${\text{P}}_{\text{i}}$ denotes the monthly precipitation (mm/a).

#### 2.3.4 Flood mitigation.

FM is the function of ecosystems to reduce flood hazards by regulating stormwater runoff and attenuating flood peaks. This study employs the water balance equation and reservoir and lake flood storage model [28] to calculate FM based on precipitation, surface runoff, and LULC.


$${\text{C}}_{\text{fm}}\text{=(P-R)×0.001×S}+\text{.35×}{\text{C}}_{\text{t}}+{\text{e}}^{\text{4.924}}\text{×}{\frac{\text{S}}{{\text{10}}^{\text{6}}}}^{\text{1.128}}\text{×3.19}$$ (4)

where ${\text{C}}_{\text{fm}}$ is the amount of FM (m³/a); P stands for annual precipitation (mm/a); R is the yearly surface runoff (mm/a); S indicates the pixel area (m²); ${\text{C}}_{\text{t}}$ denotes the total reservoir capacity (m³).

#### 2.3.5 Climate regulation.

The ability of ecosystems to absorb energy, lower temperatures, and enhance humidity through processes such as vegetation transpiration and the evaporation from water bodies is known as CR. An evapotranspiration model is used to estimate the energy expended by ecosystems in CR, with calculations based on precipitation, temperature, and LULC [29].


$${\text{Q}}_{\text{we}}\text{=EPP }\;\;\times \;\;\text{ S }\;\;\times \;\;\text{ D }\;\;\times \;\;\;\frac{\;\;\;\omega \;\;\;}{\text{r}}+0.001\text{E }\;\;\times \;\;\text{ S }\;\;\times \;\;\text{ ( }\;\;\rho \;\;\;\;\;\times \;\;\text{ q }\;\;\times \;\;\;\;\;\omega \;\;\text{ +y)}$$ (5)

where${\text{Q}}_{\text{we}}$ represents the energy expended by ecosystems in CR (kWh); EPP represents the heat absorbed by vegetation per unit area (kJ/m²·d), The heat absorbed by evapotranspiration from forest, shrubland, and grassland is taken as 2837.27, 1300.95, and 969.83 kJ/m²·d, respectively; D denotes how many days the daily maximum temperature rises over 26°C; S refers to the pixel area (m²);  ω  is a constant (kWh/kJ), equal to $\frac{\text{1}}{\text{3600}}$, and r is the coefficient of performance (COP) of the air conditioner, taken as 3; E represents the annual evaporation (mm); ρ denotes the density of water (kg/m³), equal to 1000; and q refers to the latent heat of vaporization of water at standard atmospheric pressure (kJ/kg), equal to 2260; y stands for the electricity usage of 125 kWh needed by the humidifier to turn 1 m³ of water into steam.

### 2.4 Land use transition matrix and ESs of various ecosystems

A tangible illustration of the interactions between human activity and the environment can be found in changes in ecosystem structure. The land use transition matrix (LUTM) is employed in this study as a quantitative method to evaluate the spatial changes in various ecosystems over time, while also identifying the trends and structural patterns of these alterations. The formula for the LUTM is as follows:


$${\text{A}}_{\text{ij}}\text{=}[\begin{array}{cccc} {\text{A}}_{\text{11}} & {\text{A}}_{\text{12}} & \cdots & {\text{A}}_{\text{1n}}\\ {\text{A}}_{\text{21}} & {\text{A}}_{\text{22}} & \text{…} & {\text{A}}_{\text{2n}}\\ \vdots & \vdots & \ddots & \vdots \\ {\text{A}}_{\text{n1}} & {\text{A}}_{\text{n2}} & \ldots & {\text{A}}_{\text{nn}} \end{array}]$$ (6)

where A is the land area (m²); ${\text{A}}_{\text{ij}}$ refers to the area undergoing conversion from land use category i to category j; n signifies the total count of land use categories, with i and j indicating the land use types prior to and following the transition, respectively.

To address the differences in units of measurement for ESs and ensure data comparability, this study utilized the min-max normalization technique to remove dimensional effects. Subsequently, based on land use data from the HJRB, the zonal statistics toolbox in ArcGIS is utilized to calculate ESs for various ecosystem types. This analysis facilitates a deeper exploration of the impacts of IBWT on ESs, particularly the spatiotemporal variation characteristics of ESs across different ecosystem types.

### 2.5 Patterns of ESs importance change

In this study, the research area is divided into four zones based on the volume of ESs: Non-important area (N), Low-important area (L), Moderately important area (M), and Highly important area (H). To further analyze the spatiotemporal evolution characteristics of ESs under the context of IBWT, an importance zone transition matrix was constructed. Table 2 integrates three key types of information: (1) importance zone types (N-L-M-H classification system); (2) importance fluctuation (reflecting the trends of ESs changes over time and space, i.e., increase or decrease); and (3) changes in importance zones (quantifying the transition relationships between different importance zones). This table reveals the evolution patterns of ESs across different temporal and spatial scales and provides quantitative support for analyzing the impacts of IBWT on water source areas.

**Table 2:** Regionalization standard of ESs importance change pattern.

Types of Important Areas	Fluctuation of Importance	Changes in Important Areas	Abbreviation
N	Increase	N transforms into L	N-L
		N transforms into M	N-M
		N transforms into H	N-H
L	Decrease	L transforms into N	L-N
	Increase	L transforms into M	L-M
		L transforms into H	L-H
M	Decrease	M transforms into N	M-N
		M transforms into L	M-L
	Increase	M transforms into H	M-H
H	Decrease	H transforms into N	H-N
		H transforms into L	H-L
		H transforms into M	H-M

## 3. Results

### 3.1 Ecosystem structure and changes

**Fig 3:**
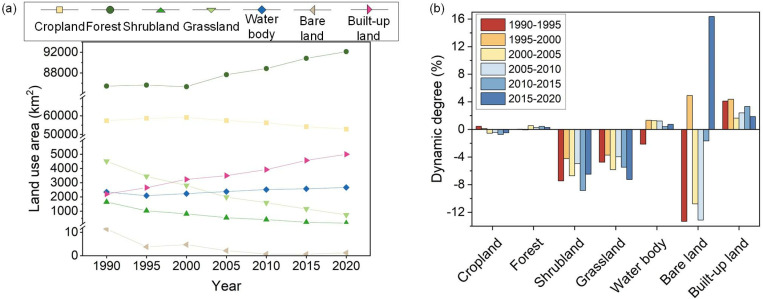
Trend of changes in various ecosystems from 1990 to 2020 (a); Dynamics of various ecosystems in different periods (b).

Based on the analysis of trends in various ecosystem types from 1990 to 2020 (Fig 3a), the HJRB encompasses diverse ecosystem types, with each exhibiting distinct area trends. Notably, forest and water body areas have shown an increasing trend, whereas built-up land has persistently expanded. Conversely, shrubland and grassland areas have rapidly decreased, and cropland areas have also declined. These changes are closely and intricately linked to the implementation of the MR-SNWD, manifesting as significant shifts in ecosystem structure across different phases.

Before the project initiation (1990–2000), land use in the HJRB was dominated by agricultural expansion. Cropland area increased by 1,740.04 km², primarily due to the conversion of 5.09% forest, 4.76% shrubland, and 14.71% grassland into cropland, concentrated around the main stream of the Hanjiang River. Concurrently, 1.59% of cropland was transformed into built-up areas, leading to an expansion of 1,032.88 km² in built-up land, reflecting an annual growth rate of 4.69% (Fig 3b). This reflects an economic development model during this period that prioritized agricultural production and infrastructure construction.

**Fig 4:**
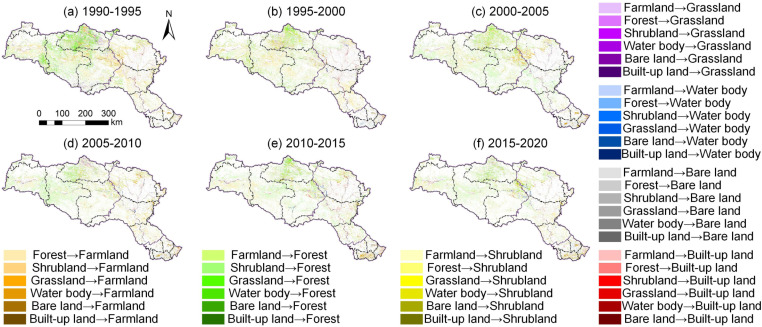
Conversion of various ecosystems at different time periods.

After the project initiation (2000–2020), the ecosystem structure of the HJRB underwent significant transformation (Fig 4). Forest area continued to increase, rising by 6,788.59 km², with 12.26% of cropland, 83.69% of shrubland, and 50.88% of grassland converted into forest, primarily in the upstream and midstream forested regions. Although 3.07% of cropland was still converted into built-up land, resulting in a 1,763.31 km² increase in built-up land area, the annual growth rate decreased to 2.73%, significantly lower than the pre-project level. This shift indicates that the implementation of the MR-SNWD and its associated ecological conservation policies effectively promoted vegetation restoration while curbing urban expansion, particularly in the midstream region. Notably, during the project operation phase (2010–2020), changes in water body area were particularly striking. As the water diversion volume increased from 2.16 billion m³ to 8.622 billion m³, the water body area of the Danjiangkou Reservoir expanded from 474.69 km² to 731.94 km², a 54.19% increase. However, water body areas in the midstream and downstream regions decreased by 153.16 km², an 8.64% reduction, highlighting the significant impact of IBWT on the spatial distribution of water resources.

### 3.2 ESs of various ecosystems

**Fig 5:**
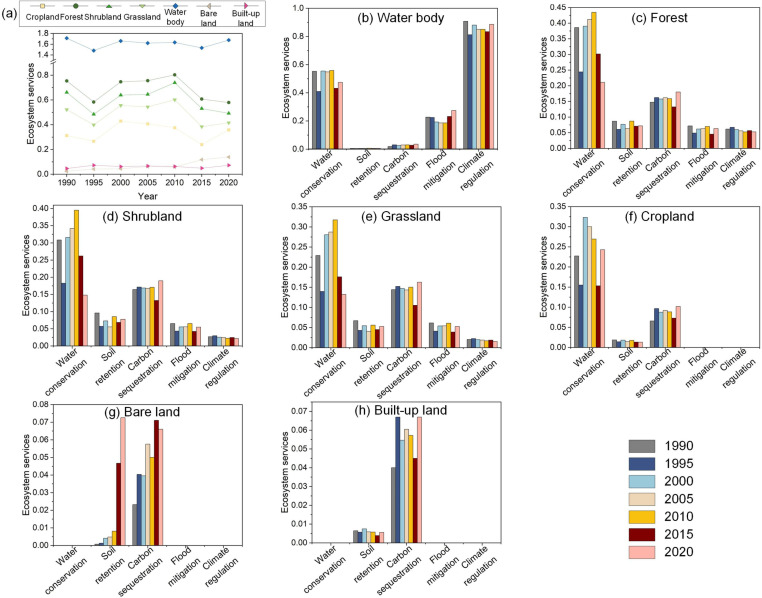
ESs of various ecosystems from 1990 to 2020.

From 1990 to 2020, the ESs provided by various ecosystems in the HJRB exhibited significant temporal variations (Fig 5). Water bodies maintained relatively stable and high levels of ESs throughout the period, primarily offering WC, FM, and CR, with a fluctuation of only 2.06%. Forests, shrublands, and grasslands, as key ecosystem types, also provided multiple ESs, mainly WC, SR, and CS. However, between 2010 and 2020, the ESs of forests, shrublands, and grasslands declined significantly, with decreases of 27.89%, 33.40%, and 30.92%, respectively. Croplands also contributed to WC, SR, and CS to a certain extent. Notably, the ESs of croplands decreased from 0.38 to 0.24 between 2010 and 2020, and then rebounded to 0.36. In contrast, bare lands and built-up lands had lower levels of ESs, making limited contributions to the overall ESs.

### 3.3 Spatial distribution of ESs

**Fig 6:**
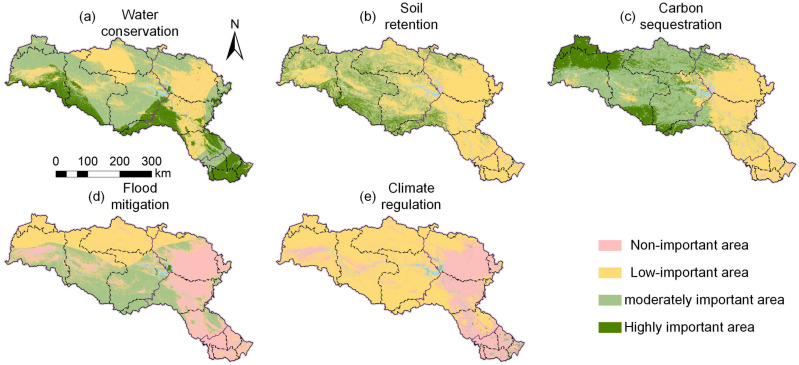
The HJRB’s spatial distribution of ESs.

The regional distribution of ESs exhibits significant heterogeneity (Fig 6). The Danjiangkou Reservoir, as the core water source of the MR-SNWD, plays a crucial role in FM, WC, and CR. Its water storage capacity provides a stable water source for the basin, and through evaporation, it effectively increases air humidity, regulates temperature, and has a positive impact on the local climate. The southern mountainous areas of the basin, due to their higher vegetation coverage, abundant precipitation, complex terrain, and stable soil structure, serve as Highly important areas for WC and SR. Additionally, the southern and northwestern mountainous regions of the basin, with their higher altitudes and abundant forest resources, exhibit significant CS. Overall, the Danjiangkou Reservoir, along with the southern and northwestern mountainous areas of the basin, plays an essential role in maintaining ESs in the HJRB.

### 3.4 Changes in ESs over space and time

**Fig 7:**
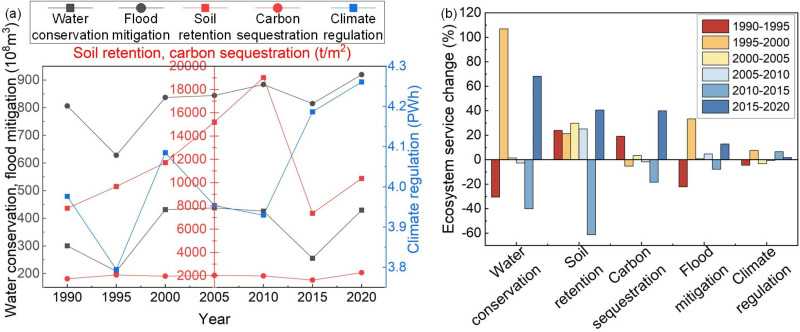
Trend of changes in ESs from 1990 to 2020 (a); Magnitude of changes in ESs during different periods (b).

**Fig 8:**
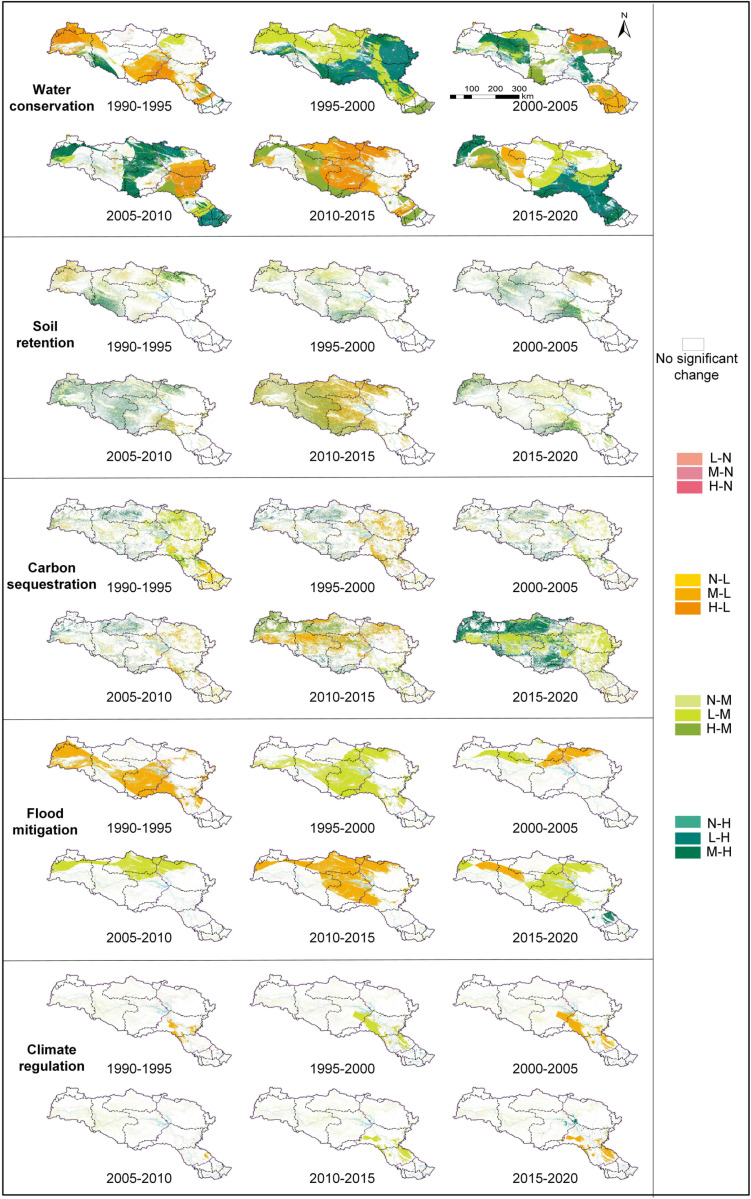
Relative changes in ESs during different periods.

According to the analysis of the trends and magnitudes of ESs changes (Figs 7 and 8), from 1990 to 2000, WC and FM exhibited significant fluctuations, showing a sensitive response to changes in precipitation. From 2000 to 2005, there was an increase in WC, SR, CS, and FM, with increases of 6.28 × 10^8^ m³, 3,487.81 t/m², 67.04 tCO₂/m², and 6.94 × 10^8^ m³, respectively, primarily concentrated in the upstream region. However, after the Danjiangkou Dam heightening project (2005–2010), WC and CS declined, with reductions of 12.02 × 10^8^ m³ and 36.28 tCO₂/m², primarily in the midstream region. Between 2010 and 2015, as the MR-SNWD initiated its first water diversion, ESs across the entire basin underwent a significant decline. WC decreased by 170.37 × 10^8^ m³, SR by 11,651.50 t/m², CS by 367.93 tCO₂/m², and FM by 68.95 × 10^8^ m³. From 2015 to 2020, WC and SR began to recover, increasing by 174.14 × 10^8^ m³ and 2,992.28 t/m², respectively, mainly concentrated in the core water source area of Shiyan, as well as the midstream and downstream regions. CS improved, increasing by 649.71 tCO₂/m², primarily concentrated in the upstream and midstream regions. At the same time, FM and CR increased by 104.94 × 10^8^ m³ and 0.07 PWh, respectively, with significant concentration in the Danjiangkou Reservoir.

## 4. Discussion

### 4.1 Impact on ecosystem structure of project initiation

The spatiotemporal dynamics of the ecosystem structure in the HJRB between 1990 and 2020 are thoroughly examined in this article. The findings demonstrate that the MR-SNWD’s implementation has profoundly influenced the HJRB’s ecosystem, with significant variations in these impacts across different phases.

Before the project initiation (1990–2000), the ecological space in the HJRB was heavily encroached upon by production space. Specifically, large areas of forest, shrubland, and grassland were converted into cropland, while portions of cropland were further transformed into built-up land. This transformation reflects the widespread presence of an extensive development model in many regions of China, which is driven by scale and efficiency, emphasizing large-scale land development and resource utilization, and often resulting in negative environmental impacts [30].

During the project initiation phase (2000–2010), the ecosystem structure of the HJRB has shown significant improvement. Specifically, the conversion of cropland, shrubland, and grassland into forest promoted an increase in forest area, leading to enhancements in ESs such as SR, CS, and FM. This result contrasts with previous studies, which have suggested that after the initiation of IBWT, ecosystems such as cropland, grassland, and forests in water source areas are often temporarily or permanently occupied, leading to reduced biodiversity and threatening ESs [31]. The observed difference primarily results from the concurrent implementation of ecological restoration efforts in the HJRB and the initiation of the MR-SNWD [32]. This finding suggests that the simultaneous implementation of ecological restoration and large-scale infrastructure projects can effectively counteract the negative ecological impacts of such projects while facilitating the restoration of ecosystem functions.

During the project operation phase (2010–2020), with the increase in water diversion volume, the water body area of the Danjiangkou Reservoir expanded significantly, whereas the water body area in the Hanjiang River’s midstream and downstream sections decreased. This finding aligns with existing studies [33], indicating that the water diversion project, through human intervention in hydrological processes, has caused an increase in water storage areas upstream and the shrinkage of natural water bodies downstream, highlighting the project’s strong interference with the water cycle. Additionally, the annual growth rate of built-up land in the HJRB slowed significantly, particularly in the midstream region. This phenomenon is primarily attributed to two reasons: First, the water diversion project reduced overall water resources in the Hanjiang River’s midstream, compelling local governments to strengthen water resource management and restrict high water-consuming industries and extensive urban expansion [34]. Second, the enforcement of ecological preservation measures, including the delineation of water source protection zones and the Grain for Green Program, further reduced the available space for built-up land [35].

### 4.2 Impact of dam heightening and project operation on ESs

There are significant spatial and temporal variations in the effects of IBWT on the ESs of water source areas. The results of this study show that between 2005 and 2010, the WC and CS in the midstream region of the HJRB experienced a noticeable decline. This was primarily due to the Danjiangkou Dam heightening project, which altered the watershed’s hydrological cycle and water processes, leading to reduced river flow in the midstream and downstream regions and thereby impacting the regional ecosystem’s health and stability [36]. This aligns with prior research [37], indicating that dam construction can interfere with watershed hydrological processes, potentially causing the degradation of ecological functions, especially in areas with restricted water flow. After 2010, with the increased storage capacity of the Danjiangkou Reservoir, the reservoir’s FM and CR significantly improved. The enlargement of the reservoir enhanced its ability to regulate heavy rainfall and floods, effectively absorbing excess water and reducing the risk of downstream flooding. At the same time, the reservoir’s evaporation and heat exchange processes played a positive role in stabilizing the regional climate [38]. This result aligns with existing research [39], further confirming the critical ecological role of the reservoir in FM and CR.

During the initial phase of the project (2010–2015), the MR-SNWD caused considerable adverse effects on the HJRB’s cropland, forest, shrubland, and grassland ESs, resulting in substantial reductions in WC, SR, CS, and FM. The main factor contributing to this was the shift in water and soil moisture distribution caused by the IBWT, which disturbed the balance of water and energy between the atmosphere and the land surface [40]. This result is consistent with previous studies [41], which indicate that IBWT operation significantly impairs the ecological functions and stability of water source ecosystems. Particularly under conditions of insufficient precipitation, the initial implementation of ecological restoration measures failed to effectively mitigate the ecological pressures caused by water diversion, and in some cases, even exacerbated the degradation of ecological functions [42]. This result further emphasizes that IBWT may intensify the degradation of ESs in water source areas under unfavorable climatic conditions. During the sustained operation phase (2015–2020), the project’s impact on ESs exhibited significant regional differences. In the upstream region (e.g., Hanzhong City and Ankang City), WC and FM further weakened, primarily due to the continuous outflow of water resources reducing water supply and thereby limiting ecosystem recovery capacity [43]. This process demonstrates that the rise in water diversion volume has intensified water resource pressure in the upstream region, exacerbating the negative impacts on ESs. In contrast, in the midstream and downstream regions, where precipitation is relatively abundant, ecological restoration measures played a crucial role under favorable climatic conditions [44], promoting improvements in WC, SR, and CS.

The ecological impacts of IBWT are closely related to seasonal hydrological characteristics. Water diversion during dry seasons may exacerbate water scarcity in upstream region, leading to vegetation water stress and subsequently weakening ecological functions. In contrast, water diversion during wet seasons, while mitigating flood risks downstream through reservoir flood retention effects, may disrupt natural hydrological rhythms, potentially inhibiting material cycling and biodiversity maintenance in floodplain areas [45]. This seasonal contradiction is particularly pronounced in monsoon regions like India, where uneven water resource distribution between wet and dry seasons has become a critical challenge for IBWT [46]. Future studies need to integrate hydrological models and ecological monitoring to quantify the relationship between water diversion intensity and ecosystem responses across different seasons. Additionally, IBWT may trigger cross-regional migration of pollutants. For instance, in China’s Bohai Bay region, studies have shown that water diversion may exacerbate the long-distance migration of persistent organic pollutants [47]. Simultaneously, microplastics and their adsorbed heavy metals may also enter receiving areas through water diversion, posing threats to regional ecological security [48]. Future research should incorporate pollutant tracking technologies to systematically evaluate the potential effects of water diversion initiatives on water quality security.

### 4.3 Impact of ecological restoration on ESs

**Fig 9:**
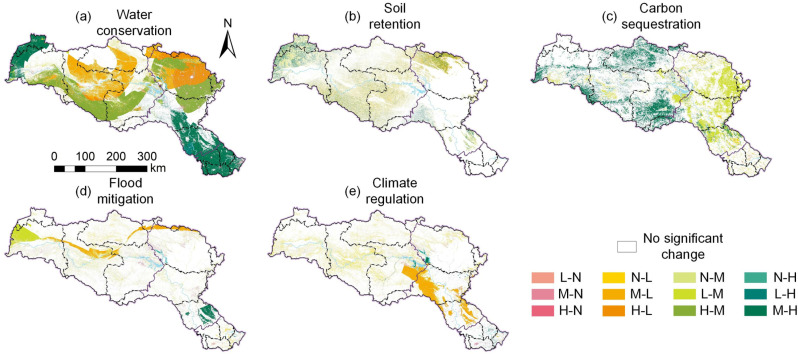
Relative changes in ESs from 2000 to 2020.

To reduce the adverse effects of the MR-SNWD on the ecological environment of water source areas, the government introduced the “Water Pollution Prevention and Soil Conservation Plan for the Danjiangkou Reservoir Area and Its Upstream” within the HJRB, achieving significant results. The upstream and midstream areas of the HJRB have seen notable improvements in CS due to various ecological restoration programs, including the Natural Forest Protection Initiative, the Grain for Green Program, and the Soil Conservation Strategy [49]. Meanwhile, WC, SR, and FM have been improved in the core water source area of Shiyan and the midstream region (Fig 9). These outcomes align with global experiences in ecological restoration. For instance, ecological restoration projects in central Arizona, USA, and California’s Sacramento-San Joaquin River Delta have achieved significant success [50,51], demonstrating the critical role of ecological restoration in rehabilitating water source ecosystems. These successful examples underscore the critical contribution of ecological restoration measures in alleviating the adverse effects of IBWT on the ecosystems of water source regions.

However, the WC, SR, and FM in the upstream region and the core water source area of Nanyang have not yet been fully restored, despite the HJRB’s ecological environment having improved. These issues may be closely related to differences in local governance capacity, changes in hydrological conditions, and inadequacies in water allocation plans [52].

### 4.4 Impact of the YHWD on ESs

From 2015 to 2020, the ecological structure in the downstream region of the HJRB underwent significant changes, primarily characterized by the shift of forests and water bodies to cropland, and the conversion of cropland to built-up land. These changes typically result in a decline in ESs [53]. However, this study found that WC and SR in the downstream region have improved, primarily because of the YHWD’s implementation. This initiative has enhanced water availability in the Hanjiang River’s downstream region, boosted ecological flow, and thereby facilitated the restoration and safeguarding of the ecosystems [54]. These findings indicate that the YHWD has played a crucial role in regulating the ecological balance and enhancing ESs in the downstream region of the HJRB, serving as a key strategy to mitigate the negative ecological impacts of the MR-SNWD on this area’s ecological environment.

### 4.5 Recommendations for ecosystem protection in the HJRB

This study analyzes the ESs of the HJRB over the past 30 years, revealing that vegetation and water bodies play significant roles in WC, SR, CS, FM, and CR. Existing research indicates that alterations in the extent of vegetation and water bodies have a direct impact on the integrity and connectivity of ecosystems [55]. Based on this finding, it is recommended that the government adopt a comprehensive set of ecological protection strategies to strengthen the management and protection of these critical ecological elements. Prioritization should be given to safeguarding the mountainous areas in the southern and northwestern regions of the HJRB, along with the Danjiangkou Reservoir, which are crucial for sustaining ESs. This demands boosting forest resource preservation and management in these locations, promoting the restoration and renewal of forest vegetation, and enhancing the monitoring and prevention of pest and disease outbreaks. Simultaneously, the management and operational standards of Danjiangkou Reservoir should be improved, emphasizing water quality monitoring and control, as well as the rational use and protection of water resources, and developing a scientifically informed hydrological scheduling plan for the reservoir.

This research emphasizes the enduring effects of IBWT on ESs in water source areas, particularly in the upstream region and the core water source area of Nanyang, where WC, SR, and FM have not been fully restored. Based on this finding, future water resource management policies should place greater emphasis on the sustainability of ecosystems, ensuring a coordinated development between water resource use and ecological preservation. In light of this, this study proposes four key recommendations: First, promote the development of comprehensive water transfer plans to ensure a balance between the rational exploitation and utilization of water resources and ecological protection; second, establish and improve ecological compensation mechanisms to effectively offset the loss of ESs; third, strengthen regional coordination and governance mechanisms to promote cross-regional cooperation and information sharing; and finally, conduct long-term ecological monitoring and assessment to ensure the timely identification and response to potential risks posed by IBWT to the ecological environment. By adopting these measures, the adverse effects of IBWT on the ecological environment can be effectively reduced, thus promoting the long-term stability and functional restoration of ecosystems.

Furthermore, the socio-cultural impacts of IBWT cannot be overlooked. Studies indicate that such projects may lead to a series of social issues, including resettlement, changes in traditional livelihoods, and conflicts over cultural heritage preservation [56]. In line with the United Nations Sustainable Development Goals (SDG 6.5), water resource management should fully consider social inclusivity and protect ecological landscapes closely tied to cultural services [57]. Therefore, it is recommended to integrate socio-cultural assessments into ecological compensation mechanisms and mitigate the potential negative impacts of water diversion projects on local social structures through community participation and cultural heritage preservation.

### 4.6 Restrictions and upcoming work

Although this study has provided some insightful information, there are still a number of drawbacks. Firstly, the existing assessment models for ESs have considered a limited number of variables, which may lead to biased results. For example, in the evaluation of WC, the influence of topographical features has not been adequately accounted for, while the importance of precipitation in the evaluation of CR has been insufficiently emphasized, causing temperature variations to disproportionately affect the model outcomes. Therefore, future research should adopt more refined models for analysis. Second, this study concentrated solely on the influence of IBWT on regulating services, without taking into account the project’s total ecological impact on the water source area. Future studies should widen their scope to investigate the whole impact of IBWT on supporting services (e.g., habitat quality), provisioning services (e.g., food production, water supply), and cultural services (e.g., landscape aesthetics) in water source locations. Additionally, due to limitations in data sources and availability, there is a lack of validation for the results of the ESs assessment. Subsequent studies should aim to obtain more high-resolution remote sensing data and field observation data to improve the precision of the findings.

Despite the aforementioned limitations, this study has still revealed the spatiotemporal distribution characteristics of the ecosystem structure and ESs in different stages of the HJRB and highlighted the impact of the MR-SNWD on the ESs of the basin. Based on these findings, future research could delve into the following directions: (1) analyzing the impacts of seasonal variations in water quantity and quality on ESs in water source areas within IBWT and investigating the potential risks of pollutant transfer from water source areas to receiving areas; (2) analyzing the interrelationships between different ESs to assess the comprehensive impact of IBWT on ecosystems; (3) identifying the ecosystem service bundles of water source areas and exploring the dynamic changes of these bundles during the inter-basin water transfer process; (4) integrating future climate change and land use change scenarios to simulate forecasts and evaluate their potential effects on ESs within the framework of IBWT.

## 5. Conclusion

This work examines the spatiotemporal changes in the ecosystem structure and its primary ESs (such as WC, SR, CS, FM, and CR) in the HJRB from 1990 to 2020, utilizing the MR-SNWD as a case study. The study further explores the impacts of project initiation, the dam heightening, project operations, and ecological restoration measures on ESs in the basin. The aim is to assess the long-term consequences of IBWT on the ESs of water source areas and their spatiotemporal evolution. The key findings of this study are as follows:

(1) Synchronous implementation of ecological restoration and engineering projects can effectively alleviate ecological pressures. From 2000 to 2010, forest coverage in the HJRB rose by 4.12%, while SR, CS, and FM improved by 62.32%, 1.57%, and 5.58%, respectively. This demonstrates that combining ecological restoration measures and infrastructure construction can significantly reduce the adverse ecological effects of engineering projects and establish a basis for restoring ecosystem functions.
(2) The impact of dam heightening on ESs exhibits spatiotemporal heterogeneity. From 2005 to 2010, the Danjiangkou Dam heightening project led to a 2.75% reduction in WC and a 1.79% decline in CS in the midstream region of the HJRB. However, with the increase in reservoir capacity, the Danjiangkou Reservoir showed significant improvements in FM and CR from 2010 to 2020, with increases of 4.07% and 8.43%, respectively. These results reveal the reshaping mechanisms of dam heightening on ES supply patterns.
(3) Project operation has significant negative impacts on ESs. From 2010 to 2015, the ESs in cropland, forest, shrubland, and grassland in the HJRB declined by 36.29%, 24.38%, 28.29%, and 36.20%, respectively. Specifically, WC decreased by 40.02%, and SR reduced by 61.25%. With the increase in water diversion volume, ESs in upstream regions (e.g., Hanzhong City and Ankang City) further weakened, highlighting the long-term pressure of project operation on the water source ecosystem.
(4) Ecological restoration efforts have been instrumental in alleviating the adverse effects of the project. From 2000 to 2020, CS was enhanced in the HJRB’s upstream and midstream regions. Additionally, WC, SR, and FM in the core water source area of Shiyan and the midstream region were improved. However, ESs in the upstream region and the core water source area of Nanyang have not fully recovered. These results validate the dynamic interplay between ecological restoration and project intervention, showing that ecological restoration can partially mitigate the negative impacts of the project, but the effectiveness varies across regions due to local conditions. Therefore, when formulating ecological restoration and management strategies, it is imperative to thoroughly evaluate the distinct conditions of each region and employ customized methods.

In summary, this study systematically assesses the impacts of IBWT on ESs throughout their lifecycle, addressing the limitations of existing studies that often focus on a single phase or localized region. It reveals the spatiotemporal heterogeneity of the project’s impacts on ESs and proposes management strategies based on the dynamic relationship between ecological restoration and project intervention, providing a scientific basis for optimizing the balance between water resource redistribution and ecological protection. The results of this research hold substantial practical relevance for the sustainable management of IBWT, offering scientific support for policymakers and serving as a reference for ecological impact assessments and sustainable management of similar projects.
